# Hydraulic Transients in the Long Diversion-Type Hydropower Station with a Complex Differential Surge Tank

**DOI:** 10.1155/2014/241868

**Published:** 2014-07-15

**Authors:** Xiaodong Yu, Jian Zhang, Ling Zhou

**Affiliations:** ^1^College of Water Conservancy and Hydropower Engineering, Hohai University, Nanjing 210098, China; ^2^College of Hydraulic and Civil Engineering, Xinjiang Agricultural University, Urumqi 830052, China

## Abstract

Based on the theory of hydraulic transients and the method of characteristics (MOC), a mathematic model of the differential surge tank with pressure-reduction orifices (PROs) and overflow weirs for transient calculation is proposed. The numerical model of hydraulic transients is established using the data of a practical hydropower station; and the probable transients are simulated. The results show that successive load rejection is critical for calculating the maximum pressure in spiral case and the maximum rotating speed of runner when the bifurcated pipe is converging under the surge tank in a diversion-type hydropower station; the pressure difference between two sides of breast wall is large during transient conditions, and it would be more serious when simultaneous load rejections happen after load acceptance; the reasonable arrangement of PROs on breast wall can effectively decrease the pressure difference.

## 1. Introduction

Various turbine operations such as load acceptance, load rejection, and combination conditions produce transients in hydropower station, which are directly related to the safety of whole hydropower station and local power grid, even resulting in substantial damage and human loss in some cases [1–3]. Study of hydraulic transients in hydropower stations attracts many researchers because of its complexity and significance in practice. Souza et al. [4] simulated transient flow in hydropower plants by considering a nonlinear model of the penstock and hydraulic turbine. Selek et al. [5] compared the computational results of transient pressures with measured data for the Catalan Power Plant in Turkey and the agreement is satisfactory. Calamak and Bozkus [6] studied the performance of two run-of-river plants during transient conditions. An et al. [7] presented an effective theory for safe control of air cushion surge chambers based on the numerical simulation of hydraulic transients in hydropower station. The impulse method was used to analyze hydraulic resonance in hydropower systems by Riasi et al. [8]. Except for the MOC [9], some new numerical methods have been used to simulate hydraulic transients in hydropower systems, such as dynamic orifice model [10], finite volume method [11], implicit method of characteristics [12], stochastic method [13], and 1-D-3-D coupling approach [14].

With the rapid exploitation of hydropower resources in some developing countries in recent years, as well as the advanced construction techniques, the arrangement of the pipe systems and hydraulic devices is more complicated. Some diversion-type hydropower stations have a fairly long headrace tunnel, and the water inertia is very large. In order to reduce the amplitude of water level oscillations in surge tanks and accelerate the attenuation, a new type of differential surge tank [15, 16] is designed. However, some undesirable transients may appear because of the complex arrangements and new hydraulic devices [17].

This work investigates the transients in a practical hydropower station with long headrace tunnel and complex differential surge tank. Some undesirable transients are mainly analyzed and the PROs are proposed to reduce the pressure difference on the breast wall. The results can provide reference for the design and operation of a similar project.

## 2. Mathematic Model and Calculation Method

### 2.1. Governing Equations and Solution Methods for Pressurized Pipe Model

The following equations describe one-dimensional transient flows [18].

 Continuity equation:
  (1) $$\begin{array}{c} \frac{\partial H}{\partial t}+V\frac{\partial H}{\partial x}+\frac{a^{2}}{g}\frac{\partial V}{\partial x}+V\sin\theta =0. \end{array}$$
 Momentum equation:
  (2) $$\begin{array}{c} \frac{\partial H}{\partial x}+\frac{V}{g}\frac{\partial V}{\partial x}+\frac{1}{g}\frac{\partial V}{\partial t}+\frac{f\left|V\right|V}{2gD}=0, \end{array}$$

in which *H* = piezometric head, *V* = flow velocity, *a* = wave speed, *g* = acceleration due to gravity, *f* = Darcy-Weisbach friction factor, *θ* = angle of the pipeline inclined with the horizontal, *x* = distance along the pipeline measured positively in the downstream direction, *D* = diameter of the pipe, and *t* = time.

The momentum and continuity equations governing transient flow in closed conduits are classified as quasilinear hyperbolic partial differential equations (PDEs) for which no analytical solutions are available for a general pipe system. The method of characteristics (MOC) is widely employed to solve the set of PDEs and these two equations can be transformed into a couple of ordinary differential equations (ODEs) along the characteristic lines. The friction item is linearized by using the trapezoidal rule which is of second-order accuracy and some small terms are dropped without loss in accuracy. It is convenient to develop these equations with the discharge *Q* instead of flow velocity *V* as the dependent variable. Then, the equations can be transformed into two algebraic equations for computer calculation after integration along the characteristic lines. The grid of pipelines and the time interval follow Courant condition, Δ*t* ≤ Δ*x*/*a*.

### 2.2. Turbine Equations

The equation for the acceleration of the rotating masses is [19]

(3) $$\begin{array}{c} I\frac{d\omega }{dt}=T-T_{g}, \end{array}$$

in which *I* = the polar moment of inertia of rotating fluid and mechanical parts in the turbine-generator combination (*I* = *WR* ^2^/*g*, where *W* = weight, *R* = radius of gyration), *ω* = the angular speed of the unit, *T* = the torque produced by water flowing through the unit, and *T*_*g*_ = the resistant torque from the generator.

When load rejection occurs, the unit is instantly disconnected from the system and, thus, becomes isolated (i.e., *T*_*g*_ = 0). Then, the unit has to be quickly closed according to the emergency closure law which is set to the governor in advance to prevent extended periods of high overspeed, which may induce severe spiral case pressure increments and draft tube pressure decrements. This type of regulation is much severe and critical to the safety of the whole system, which is considered in this study. Equation (3) can be transferred to algebraic equations by integration and Taylor expansion method, which can be expressed as

(4) $$\begin{array}{c} n_{t}=n_{t0}+\frac{\Delta t}{T_{m}}\left(1.5\beta_{t0}-0.5\beta_{t0-\Delta t}\right), \end{array}$$

in which *n* = the dimensionless rotating speed ratio, *N*/*N*_*r*_, *β* = the dimensionless torque ratio, *T*/*T*_*r*_, Δ*t* = time step, and *T*_*m*_ = the mechanical starting time, defined as *T*_*m*_ = *Iω*_*r*_^2^/*P*_*r*_.

Equation (4) is explicit and the terms in the right-hand side of the equation are known at any initial condition, so the rotating speed at each time step can be calculated. Variation of the wicket gates (WG) opening during transients can be expressed as

(5) $$\begin{array}{c} y=y_{0}-\frac{\Delta t}{T_{c}}, \end{array}$$

where *y* = the dimensionless WG opening, *y*_0_ = initial dimensionless WG opening, and *T*_*c*_ = the closing time constant from full opening to closed.

The equation for the head balance of turbine is

(6) $$\begin{array}{c} H_{T}=\left(Z_{1}+\frac{p_{1}}{\gamma }+\frac{\alpha_{1}{V_{1}}^{2}}{2g}\right)-\left(Z_{2}+\frac{p_{2}}{\gamma }+\frac{\alpha_{2}V_{2}^{2}}{2g}\right), \end{array}$$

in which, *H*_*T*_ = head of turbine, *Z* = elevation of the turbine, *p* is pressure, *V* = flow velocity, *α* = the kinetic energy correction coefficient which is generally equal to 1, *γ* = unit weight of water, *g* = acceleration of gravity, and the subscripts 1 and 2 refer to the previous and latter section of the turbine, respectively.

 Positive compatibility equation of the inlet section is
  (7) $$\begin{array}{c} H_{1}=C_{P}-B_{P}Q. \end{array}$$
 Negative compatibility equation of the outlet section is
  (8) $$\begin{array}{c} H_{2}=C_{M}+B_{M}Q. \end{array}$$

**Figure 1:**
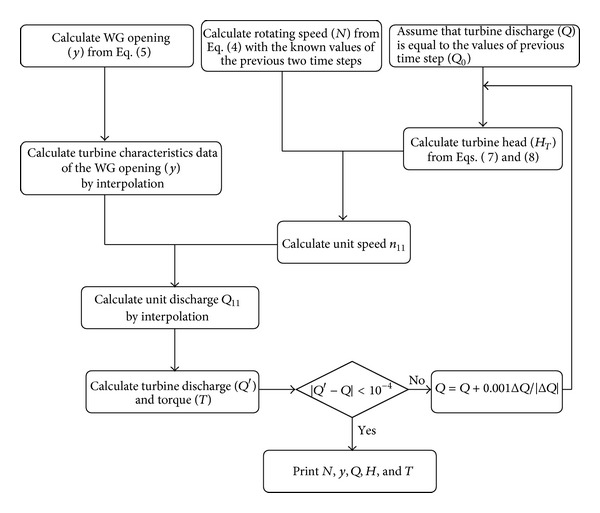
Flowchart of boundary condition for a turbine.

As is clear from the above equations, the five parameters turbine head (*H*_*T*_), discharge (*Q*), rotational speed (*N*), torque (*T*), and dimensionless WG opening (*y*) are unknown. For the calculation of these unknown values at each time step, the following procedure is used, as well as the characteristic curve of model turbine, which is schematically shown in Figure 1. The computer model is encoded in the FORTRAN programming language which has been used to simulate hydraulic transients in some practical hydropower stations and the predictions agree well with the field test [20].

### 2.3. Boundary Conditions of Differential Surge Tank with PROs and Overflow Weir

**Figure 2:**
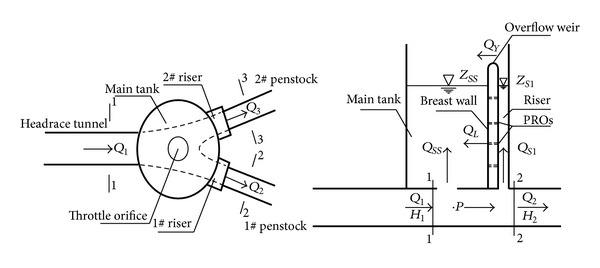
Schematic differential surge tank with PROs and overflow weir.

The plan and profile of the differential surge tank with overflow weir and PROs on the breast wall are shown in Figure 2. *H* and *Q* are the piezometric head and the discharge. The subscripts 1, 2, and 3 refer to the cross sections 1, 2, and 3, respectively. *Z*_*SS*_ and *Q*_*SS*_ are the water level and the discharge in the main tank. *Z*_*Si*_ and *Q*_*Si*_ (*i* = the number of the risers) are the water level and the discharge in the riser. *Q*_*Y*_ is the total discharge through the overflow weirs at the top of the risers. *Q*_*L*_ is the total discharge through PROs of the risers.

The mathematic models of the differential surge tank with PROs and overflow weir for transient calculation are

(9) H1+Q1Q102gA12=H2+Q2Q202gA22=H3+Q3Q302gA32=Zss+ξ1|Qss0|Qss2gATH12=Zs1+ξ2|Qs10|Qs12gATH22=Zs2+ξ3|Qs20|Qs22gATH32,Q1=Qss+Qs1+Qs2+Q2+Q3,H1=CP1−BP1Q1,H2=CM2+BM2Q2,H3=CM3+BM3Q3,dZssdt=Qss+QY+QLAss,dZs1dt=Qs1−(QY1+QL1)As1,dZs2dt=Qs2−(QY2+QL2)As2,

where, *A*_1_, *A*_2_, and *A*_3_ are the cross-sectional areas of the sections 1, 2, and 3, respectively. *A*_*ss*_, *A*_*s*1_, and *A*_*s*2_ are the cross-sectional areas of the main tank, the riser 1 and the riser 2, respectively. *A*_TH1_, *A*_TH2_, and *A*_TH3_ are the cross-sectional areas of the throttle orifices at the bottom of the main tank, the riser 1 and the riser 2, respectively. *ξ*_1_, *ξ*_2_, and *ξ*_3_ are the head loss coefficients of the throttle orifices, respectively, which have different values for the flow into or out of the tank. *Q*_*Y*_, *Q*_*Y*1_, and *Q*_*Y*2_ are the discharge through the overflow weir between the main tank and the risers, *Q*_*Y*_ = *Q*_*Y*1_ + *Q*_*Y*2_, measured positively from the risers into the main tank. *Q*_*L*_, *Q*_*L*1_, and *Q*_*L*2_ are the discharge through the PROs on the breast wall, *Q*_*L*_ = *Q*_*L*1_ + *Q*_*L*2_, measured positively from the risers into the main tank. Every parameter that has a subscript 0 is a known value of a previous time step.

Discharge through the overflow weir can be given by the equation:

(10) $$\begin{array}{c} Q_{Yi}=\{\begin{array}{cc} 0, & Z_{si}<Z_{Y},Z_{ss}<Z_{Y}\\ k_{11}B_{Y}\sqrt{2g}{\left(Z_{si}-Z_{Y}\right)}^{1.5}, & Z_{si}\geq Z_{Y},Z_{ss}<Z_{Y}\\ k_{12}B_{Y}\sqrt{2g}{\left(Z_{si}-Z_{ss}\right)}^{1.5}, & Z_{si}\geq Z_{ss}\geq Z_{Y}\\ -k_{11}^{\prime }B_{Y}\sqrt{2g}{\left(Z_{ss}-Z_{Y}\right)}^{1.5}, & Z_{si}<Z_{Y},Z_{ss}\geq Z_{Y}\\ -k_{12}^{\prime }B_{Y}\sqrt{2g}{\left(Z_{ss}-Z_{si}\right)}^{1.5}, & Z_{ss}>Z_{si}\geq Z_{Y}, \end{array} \end{array}$$

in which, *Z*_*Y*_ and *B*_*Y*_ are the elevation and the width of the overflow weir, respectively, and *k*_11_ and *k*_12_ are the discharge coefficients of free flow and submerged flow of the overflow weir, respectively.

Discharge through the PROs can be written as follows:

(11) $$\begin{array}{c} Q_{Lij}=\{\begin{array}{cc} 0, & Z_{si}<Z_{Lj},Z_{ss}<Z_{Lj}\\ \mu_{1}A_{Lj}\sqrt{2g\left(Z_{si}-Z_{Lj}\right)}, & Z_{si}\geq Z_{Lj},Z_{ss}<Z_{Lj}\\ \mu_{2}A_{Lj}\sqrt{2g\left(Z_{si}-Z_{ss}\right)}, & Z_{si}\geq Z_{ss}\geq Z_{Lj}\\ -\mu_{1}A_{Lj}\sqrt{2g\left(Z_{ss}-Z_{Lj}\right)}, & Z_{si}<Z_{Lj},Z_{ss}\geq Z_{Lj}\\ -\mu_{2}A_{Lj}\sqrt{2g\left(Z_{ss}-Z_{si}\right)}, & Z_{ss}>Z_{si}\geq Z_{Lj}, \end{array} \end{array}$$

in which *i* = 1-2, *A*_*Lj*_ (*j* = 1 − *K*) are the cross-sectional areas of the PROs, and *K* is the number of the PROs of each riser. *Z*_*Lj*_ (*j* = 1 − *K*) are the elevations of the center of the PROs, and *μ*_1_and *μ*_2_ are the discharge coefficients of free flow and submerged flow of the PROs, respectively. So the total discharge through the PROs on each breast wall may be written as

(12) $$\begin{array}{c} Q_{L1}=\overset{n}{\underset{j=1}{\sum }}Q_{L1j},\;\;Q_{L2}=\overset{n}{\underset{j=1}{\sum }}Q_{L2j}. \end{array}$$

Every variable of this boundary during transient process can be derived with (9) to (12) employing the four-order Runge-Kutta method.

## 3. Case Study

**Figure 3:**
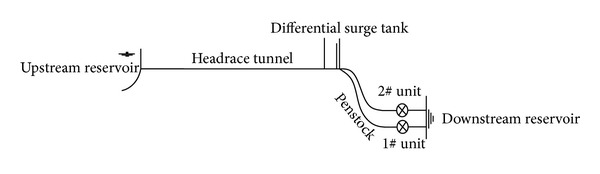
Layout of the waterway and power generation system of the hydropower station.

The model is used to predict the hydraulic transients in the practical hydropower station in China. As shown in Figure 3, every two units share a common waterway system, which consists of the intake, the gate shaft, the headrace tunnel, the upstream differential surge tank, penstock, the tailrace tunnel, and so on.

The headrace tunnel has a length of 17.0 km with a diameter *D* = 11.8 m. The differential surge tank with a “*Y*” shaped bifurcation pipe at the bottom is located at the end of the headrace tunnel. The length of each penstock is 530.0 m and the diameter is 6.5 m. The length of each tailrace tunnel is 260.0 m and the diameter is 12.0 m. The head loss coefficients of the system are collected according to the project.

The cross-sectional areas of the main tank and each riser of the differential surge tank are 346.4 m^2^ and 34.6 m^2^, respectively. The cross-sectional areas of the throttle orifice at the bottom of the main tank and each riser are 13.9 m^2^ and 19.0 m^2^, respectively. The elevation of the bottom of the main tank is 1575.2 m, while the elevation of the overflow weir at the top of the riser is 1670.0 m with the width *B*_*Y*_ = 6.0 m.

According to the laboratory experiments, the values of the parameters are as follows: the discharge coefficient of free overflow from the riser into the well *k*_11_= 0.5. The discharge coefficient of free overflow from the main tank into the riser *k*_11_′= 0.53. The discharge coefficients of submerged flow *k*_12_ and *k*_12_′ approximate to 80% of the discharge coefficients of free overflow. The head loss coefficient of the orifice at the bottom of the main tank *ξ*_1_ = 1.99 for the flow into the main tank, while *ξ*_1_ = 1.32 for the flow out of the main tank. The head loss coefficients of the orifice at the bottom of the risers *ξ*_2_ = *ξ*_3_ = 1.72 for the flow into the risers, while *ξ*_2_ = *ξ*_3_ = 3.23 for the flow out of the risers. The discharge coefficients of free flow and submerged flow of the PRO *μ*_1_ = 0.6 and *μ*_2_ = 0.5, respectively.

The rated output of each Francis turbine is 610 MW. Its rated head is 288 m, rated rotating speed is 166.7 rpm, and rated discharge is 228.6 m^3^/s. The diameter of the runner is 6.56 m. The installation elevation of turbine is 1316.8 m. The inertia (*GD* ^2^) of the turbine and generator is about 75800 t.m^2^.

### 3.1. Simulation of Simultaneous Load Rejection

**Figure 4:**
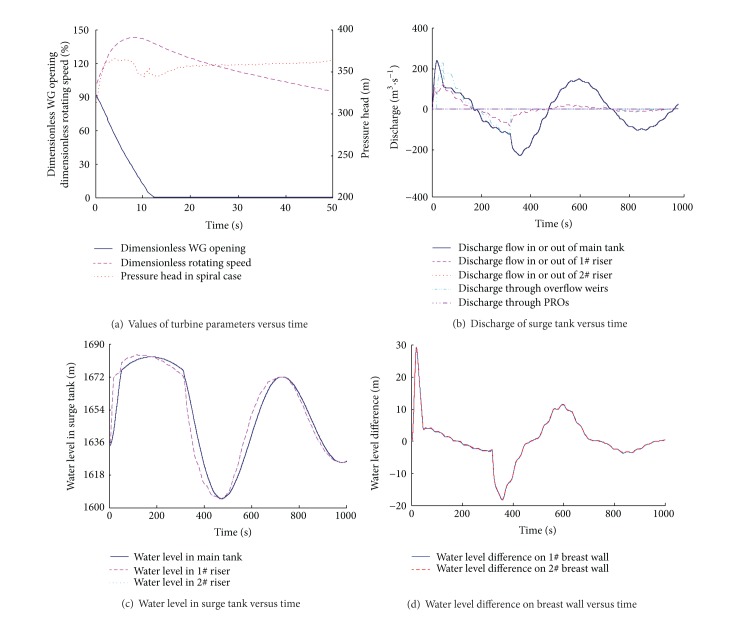
Numerical results of simultaneous load rejection.

Based on the mathematical model and numerical methods presented in the above section, the computer model of the hydraulic transients in the hydropower station is encoded in the FORTRAN programming language. A critical operation that is likely to occur several times during the life of the project is simulated. The upstream water level is at EL. 1646.0 m, and the downstream water level is at EL. 1333.1 m. Full load is rejected at time *t* = 0 and the WG are closed with the closing time *T*_*c*_ = 13 seconds under governor control. It should be noted that the effect of the PROs is ignored in this simulation; that is, the cross-section areas of the PROs are considered as 0. The calculation is shown in Figure 4.

The changes of the dimensionless rotating speed of the runner, the dimensionless WG opening, and the pressure head in the spiral case are shown in Figure 4(a). When the units reject full load, the rotating speed of the runner is increased rapidly. The WG are quickly closed according to the emergency closure law which is set to the governor in advance to prevent extended periods of high overspeed, leading to serious water hammer pressure in the spiral case. The pressure in the spiral case increases quickly during the closing process, accompanying the phenomena of the wave propagation and reflection. After the closure of the WG, most of the water hammer wave has attenuated, and the pressure in the spiral case changes slowly because of the water level variation in the surge tank.

Figures 4(b) and 4(c) show the changes of the discharge and the water levels of the surge tank. After the closure of the WG, the discharge in penstock decreases rapidly. The water in the headrace tunnel flows into the surge tank through the orifices at the bottom of the main tank and the risers, so the water levels in them increase fast. As the cross-sectional area of the riser is smaller than the main tank's, the water level in the riser rises more quickly. When the water level in the riser reaches the elevation of the overflow weir, the water freely spills into the main tank from the riser, and the increasing speed of the water level in the riser becomes slower. When the water level in the main tank reaches the elevation of the overflow weir, the free flow becomes the submerged flow. Then the discharge that flows from the riser into the main tank decreases, leading to the second quick increase of water level in the riser in a short period of time. As the discharge into the surge tank decreases, the flow direction reverses and the inflow becomes the outflow. The water levels in the main tank and the risers decrease after reaching the highest elevation. Similarly, the water level in the riser falls more rapidly because of the small cross-sectional area. When the water level in the riser is lower than that in the main tank, the water flows from the main tank to the riser, and the flow pattern changes from the submerged flow to the free flow with the variation of the water levels in the main tank and the riser. If the water level in the main tank is lower than the elevation of the overflow weir, the overflow stops and the water level in the riser falls rapidly. The discharge through the PROs is always equal to 0 during the transient because the area is set to 0 in the model.

Figure 4(d) stands for the water level difference between the two sides of the riser's breast wall during the transient process. When the load rejection happens, the water level in the riser rises quickly because of the small cross-sectional area, while the water level in the main tank increases slowly, so the corresponding pressure difference, measured positively in this situation, increases quickly. When the water level in the riser reaches the elevation of the overflow weir, it rises slowly because of the overflow, and the pressure difference reduces gradually. Then the pressure difference reverses with the decrease of the water level in the riser. Under this transient simulation, the positive pressure difference between the two sides of the riser's breast wall is about 30 m, while the negative one is about 20 m. The pressure difference is significant, so the breast wall in this kind of surge tank must be of good physical strength.

As the headrace tunnel of the hydropower station is very long, the water inertia is large, so the surge tank period is very long, the amplitude of the oscillations is large, and the attenuation is very slow. The transient process caused by previous condition has not disappeared; the following condition may happen, which may make the result of the transient more serious.

### 3.2. Simulation of Successive Load Rejection

As the layout of water diversion systems of hydropower stations becomes more complex, as well as the operation of grid systems, combination operating conditions become very common, such as load rejection after load acceptance and load rejection one by one. Although the probability of some extreme conditions is very small, once they happen, the consequences are very serious. Therefore, it is necessary to consider these factors in the design of hydropower stations.

With respect to the water hammer pressure in combination conditions, successive load rejection is studied before. This operation condition can make the negative pressure in the draft tube more serious than that in simultaneous load rejection in a pumped storage hydropower station [21]. The tailrace tunnel is short in a diversion-type hydropower station, so the draft tube pressure will not reduce too much during this condition. But successive load rejection may result in high overspeed of the runner and larger pressure in the spiral case in a long diversion-type hydropower station with the bifurcated pipe at the bottom of the surge tank; that is, there are two independent penstocks for the units from the surge tank as shown in Figure 3.

**Figure 5:**
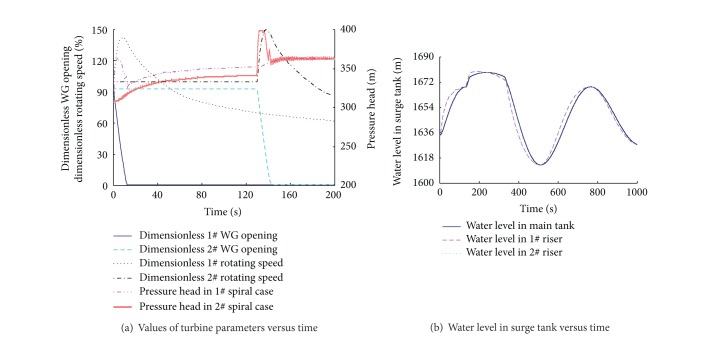
Numerical results of successive load rejection.

The calculations are shown in Figure 5. The water levels of the reservoirs are the same as the previous section. Firstly, the 1^#^ turbine rejects its full load, and when the water level of the surge tank reaches the highest level, the 2^#^ turbine rejects its full load.

The dimensionless WG opening, the dimensionless rotating speed of the runner, and the pressure head in the spiral case during successive load rejection are shown in Figure 5(a). When the 1^#^ turbine rejects full load, the water level of the surge tank begins to rise. As shown in Figure 5(b), when the water level of the surge tank reaches the highest level, it rises about 30 m, which makes the static head of the 2^#^ turbine increase by 30 m. Otherwise, the units connect with large power grid under normal conditions, and the speed of the 2^#^ turbine will not change due to the stable grid frequency, so the opening of the WG keeps constant as well. As the static head of the 2^#^ turbine rises, the demand discharge of turbine also increases. When the water level of the surge tank reaches the highest level after the load rejection of the 1^#^ turbine, the 2^#^ turbine rejects full load. Compared with simultaneous load rejection, the maximum pressure in the spiral case and the maximum rotating speed of the 2^#^ turbine are increased, which are shown in Table 1. Therefore, if the water way system of a hydropower station is arranged as this form, especially as the headrace tunnel is very long, successive load rejection condition will make the maximum pressure of the spiral case and the maximum rotating speed of the runner more serious.

### 3.3. Simulation of Simultaneous Load Rejection after Load Acceptance

Because of the small cross-sectional area, the water level in the riser rises or falls rapidly during transient process, which creates an accelerating or decelerating head on the tunnel in a short period of time. This effect reduces the amplitude of water level oscillations in the surge tank and accelerates the attenuation. However, due to rapid water level variations in the risers and slow variations in the main tank, the pressure difference between the two sides of the riser's breast wall is significant. If the structure cannot bear this pressure difference, it may cause collapse of the breast wall [22]. Thus, it is very important to find the critical operation conditions and to simulate the possible maximum pressure difference on the breast wall in the design stage, which are the basis for the structure calculation of the breast wall.

**Figure 6:**
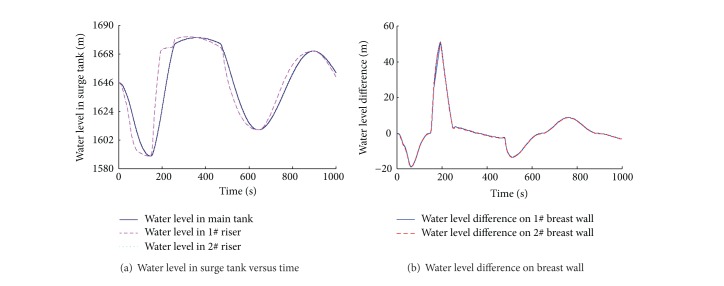
Numerical results of simultaneous load rejection after load acceptance.

Simultaneous load rejection after load acceptance is common in the operation of hydropower stations. During this simulation, the water levels of the reservoirs are the same as the previous section. As shown in Figure 6(a), two units accept load one by one, which results in the fall of the water levels in the surge tank. When the water levels of the surge tank reach the lowest level, two units reject full load simultaneously. The water levels in the risers rise rapidly to the elevation of the overflow weirs, while the water level in the main tank rises slowly and the elevation of the initial water level is lower compared with the simultaneous load rejection case. So, the pressure difference on the breast wall is larger during this combination operating condition. As shown in Figure 6(b), the maximum pressure difference on the breast wall increases by 20 m compared with simultaneous load rejection.

### 3.4. Control of the Pressure Difference on Breast Wall

As it can be seen from Table 2, the maximum pressure difference between two sides of the breast wall is close to 30 m in simultaneous load rejection, while the difference can reach 50 m in combination conditions. This huge pressure difference brings great challenge to the structural safety of the breast wall; thus, how to reduce the pressure difference between two sides of the breast wall has become an issue to the design person.

**Figure 7:**
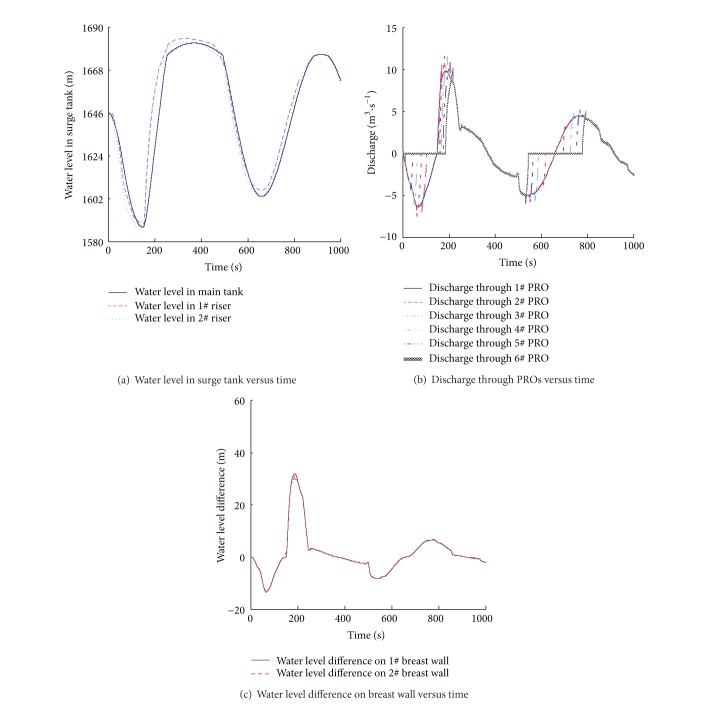
Numerical results of simultaneous load rejection after load acceptance with PROs on breast wall.

Guaranteeing adequate structural strength of the breast wall, a row of the PROs can be set along height direction [23]. When transient process occurs, a large water level difference is created between the risers and the main tank in a differential surge tank. The water level in the riser rises rapidly, reaching the PROs; then the water flows from the riser into the main tank through the orifices, which, slowing down the water level, rise in the riser while speeding the water rise in the main tank. Therefore, the pressure difference on the breast wall can be reduced. The locations, the quantity, and the diameter of the PROs are fixed during this simulation for easy comparison of the results. In this case, the elevation of the bottom floor is 1575.2 m. The first orifice is set at the elevation of 1585 m on the breast wall, with the rest PROs setting every 12 m upwards and the breast wall of each riser is installed with 6 orifices, namely, 1–6 in turn, and the diameter of each PRO is 1.0 m. The calculation condition is the same as the former section and the numerical results are shown in Figure 7.

Figure 7(a) shows the variation of the water levels in the surge tank after setting the PROs on the breast wall. Compared with the results in Figure 6(a) that no PROs are set on the breast wall, the amplitude of water level oscillations and surge attenuations in the surge tank is almost the same, while the rising speed of the water level in the riser is slowing down obviously.

Figure 7(b) shows the discharge through each PRO in the 1^#^ riser during the transients. When the units accept load, the water level falls quickly in the riser but slowly in the main tank, which forms negative water level difference on the two sides of PROs and causes the water to flow from the main tank into the riser. As the initial water levels in both the main tank and the risers are above the elevation of the highest PRO, that is, the 6^#^ PRO, submerged flow occurs in the PROs 1–6 when the water level difference appears. When the water levels in the risers fall below the elevation of the 6^#^ PRO, the flow pattern at the 6^#^ PRO turns from the submerged flow to the free flow; with the water level in the main tank continuing to fall, the discharge decreases gradually; when the water level in the main tank falls below the elevation of the 6^#^ PRO, the discharge through the 6^#^ PRO turns to 0. Similar phenomenon could be seen in other PROs when the water levels continue to fall. In addition, the flow pattern is always the submerged flow at 1^#^ PRO because of its low elevation.

When the water level reaches its lowest elevation in the surge tank, two units reject full load at the same time. In this condition, the water level in the riser rises quickly and the positive water level difference is formed in the 1^#^ PRO, causing the water to flow from the riser into the main tank in the type of submerged flow. When the water level in the riser rises to the 2^#^ PRO, the water flows from the riser to the main tank in the type of free flow; with the water level in the riser continuing to rise, the discharge through the PROs increases gradually. When the water in the main tank reaches the 2^#^ PRO, the free flow here turns to a submerged one; with the water level in the main tank continuing to rise, the discharge decreases gradually. A similar phenomenon occurs in other PROs subsequently. When the water level in the main tank is over the 6^#^ PRO, submerged flow occurs in every PRO and the discharge is the same because of the equal pressure difference on the two sides of each PRO.

Figure 7(c) shows the water level difference on the two sides of the breast wall of the riser. Because of the PROs, the water level changes slower in the riser but quicker in the main tank, which reduces the pressure difference on the breast wall of the riser. As shown in Table 2, the maximum water level difference between the two sides of the breast wall is reduced almost by 20 m, compared with the results without PROs on the breast wall. Therefore, setting appropriate PROs can effectively reduce the pressure difference between the two sides of the breast wall. It should be noted that more PROs and bigger diameters are effective to reduce the pressure difference on the breast wall, but too many or too big PROs would affect the differential effect of the surge tank, thereby increasing the maximum surge and reducing the surge attenuation in the surge tank.

## 4. Conclusions

This paper provides a mathematical model for the differential surge tank with PROs and overflow weirs for transient calculations. The numerical model of hydraulic transients is established using the data of a practical hydropower station, and the probable operation conditions are simulated and analyzed. The proposed mathematical model and the values of some coefficients used in the simulation can provide reference for the simulation of hydraulic transients in this type of hydropower station. In a long diversion-type hydropower station with the bifurcated pipe at the bottom of the surge tank, successive load rejection condition can make the maximum pressure in the spiral case and the maximum rotating speed more serious compared with simultaneous load rejection. Additionally, the pressure difference on the breast wall is significant during transients, especially during the combination condition that simultaneous load rejection after load acceptance, while setting appropriate PROs, can reduce the pressure difference effectively. Note that the present mathematical model and numerical applications need field test verification, which will be conducted in the additional investigation.

## Figures and Tables

**Table 1:** Comparisons between simultaneous and successive load rejection.

Operation conditions	Maximum pressure of spiral case	Maximum rotating speed rise
Simultaneous load rejection	367.7 m	43.2%
Successive load rejection	402.1 m	49.9%

**Table 2:** Maximum pressure difference on breast wall under different conditions.

Operation conditions	Maximum pressure difference on breast wall
Simultaneous load rejection	29.2 m
Combination conditions without PROs	50.9 m
Combination conditions with PROs	31.9 m

## Notation

MOC: Method of characteristics
ODE: Ordinary differential equation
PDE: Partial differential equation
PRO: Pressure-reduction Orifice
WG: Wicket gates
*a*: Wave velocity
*A*: Cross-sectional area of pipe
*A*_*S*_: Cross-sectional area of riser
*A*_*SS*_: Cross-sectional area of main tank
*A*_TH_: Cross-sectional area of throttle orifice
*B*_*M*_, *B*_*P*_: Known constants in compatibility equations
*B*_*Y*_: Width of overflow weir
*C*_*M*_, *C*_*P*_: Known constants in compatibility equations
*f*: Darcy-Weisbach friction factor
*GD*^2^: Moment of inertia of unit
*H*: Piezometric head
*H*_*T*_: Turbine head
*k*_11_: Discharge coefficient of free overflow through overflow weir
*k*_12_: Discharge coefficient of submerged overflow through overflow weir
*n*: Dimensionless rotating speed
*Q*: Discharge through pipe
*Q*_*L*_: Discharge through PROs
*Q*_*S*_: Discharge in riser
*Q*_*SS*_: Discharge in main tank
*Q*_*Y*_: Discharge through overflow weirs
*T*: Torque on turbine
*T*_*c*_: Closing time constant from full opening to closed
*T*_*m*_: Mechanical starting time
*y*: Dimensionless WG opening
*Z*_*L*_: Elevation of the center of PRO
*Z*_*S*_: Water level in riser
*Z*_*SS*_: Water level in main tank
*Z*_*Y*_: Elevation of overflow weir
*β*: Dimensionless torque
*ξ*: Head loss coefficient of throttle orifice
*μ*_1_: Discharge coefficients of free flow of PRO
*μ*_2_: Discharge coefficients of submerged flow of PRO.
